# Organic core–shell-shaped micro/nanoparticles from twisted macrocycles in Schiff base reaction†
†Electronic supplementary information (ESI) available: Characterization including NMR, MS, X-ray structure, SEM and TEM. CCDC 1856480 and 1856481. For ESI and crystallographic data in CIF or other electronic format see DOI: 10.1039/c8sc03824d


**DOI:** 10.1039/c8sc03824d

**Published:** 2018-10-15

**Authors:** Huaiyu Chen, Chao Huang, Yazhou Ding, Qi-Long Zhang, Bi-Xue Zhu, Xin-Long Ni

**Affiliations:** a Key Laboratory of Macrocyclic and Supramolecular Chemistry of Guizhou Province , Guizhou University , Guiyang , Guizhou 550025 , China . Email: bxzhu@gzu.edu.cn ; Email: longni333@163.com

## Abstract

Functional self-assemblies derived from noncovalent interactions such as lipid vesicles and DNA chiral double helices are a typical feature of natural life activity. Because of this phenomenon, a self-assembly approach for various functional organic particles is a desirable objective in supramolecular chemistry. Here, we report the discovery of enantiomeric conformers from a twisted macrocyclic host (**MH**), which was obtained from an achiral precursor by Schiff base reaction. Further studies suggest that a series of unexpected and stable core–shell-based organic micro/nanospheres can be directly precipitated from a simple reaction solution with high yield. A single-crystal X-ray diffraction analysis of **MH** revealed that the unusual C–H···π interaction triggered self-assembly of the enantiomeric forms in the solid state plays an important role in the formation of the core–shell-shaped organic particles.

## Abstract 2

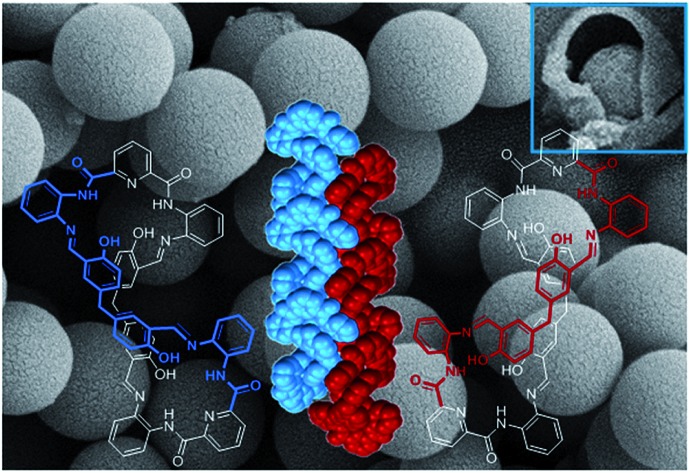
A series of organic core–shell-shaped micro/nanoparticles from twisted enantiomeric macrocycles could be obtained in Schiff base reaction with high yield at room temperature.

## Introduction

Artificial chiral macrocyclic hosts have attracted much attention because of their widespread applications in the fields of discrimination,^1^,^2^ self-assembly,^3^ catenanes,^4^ catalysis,^5^–^7^ chiral solvating agents,^8^ drug dispensers,^9^ and others. Generally, introducing chiral auxiliaries into macrocyclic skeletons is an approach for producing chiral macrocyclic hosts.^10^ In this strategy, the chiral macrocycles are easy to prepare, but the macrocyclic cavities are not fully exploited in most cases. Incorporating inherent chirality by eliminating any symmetry plane or inversion center in the cavity-shaped macrocycle skeletons is another approach to construct chiral macrocyclic hosts,^11^ but the tedious synthesis and difficulty in utilizing the macrocyclic cavities limit their practical applications. Recently, Ogoshi^12^ and Chen^13^ discovered that the linking of achiral phenol building blocks to a whole structure could provide an efficient and direct way for constructing chiral macrocyclic receptors. Our recent studies on cucurbit[*n*]urils (Q[*n*] or CB[*n*]) revealed that the large Q[*n*]s (*n* > 10) shows a strong tendency to form twisted structures.^14^,^15^ For example, twisted cucurbit[14]uril,^14^ which forms from 14 achiral glycoluril moieties and exhibits a 180° twist, gives rise to enantiomeric forms. Artificial chiral or twisted macrocyclic hosts are attractive not only because of their beautiful helical structures but also because of their unique prospects in chiral chemistry and materials chemistry.

Schiff base and related compounds, fascinating organic molecules, are often incorporated into macrocycles,^16^ cages,^17^ helices^18^ and particles^19^ as molecular building blocks because of the facile process and mild fabrication conditions. However, few examples of supramolecular self-assembly of chiral or twisted Schiff base host units have been reported. In particular, to date, no studies have fabricated pure organic nanoparticles by the self-assembly of macrocyclic hosts *via* direct organic reaction. In fact, numerous recent studies have suggested that artificial molecular chirality plays a significant role in supramolecular self-assembly processes, especially those that give rise to helical 3D superstructures.^3^ For instance, Stoddart and co-workers demonstrated interest in 3D double-helix formation by the self-sorting and self-assembly of diastereoisomeric conformations of configurationally enantiomeric macrocycles.^20^

Here, we report the discovery of enantiomeric forms from a twisted macrocyclic host (**MH**) derived from an achiral precursor by Schiff base reaction. Further studies suggest that unexpected and stable core-in-hollow-shell-based organic particles can be directly precipitated from the reaction solution with high yield. Most interestingly, the size of the core–shell-based microspheres can be further smartly tuned by the addition of different volumes of water to the reaction solution. A single-crystal X-ray diffraction analysis of **MH** revealed that the self-assembly processes of the enantiomeric forms in the solid state play a key role in the formation of such core–shell-based nanoparticle structures.

## Results and discussion

**Scheme 1:**
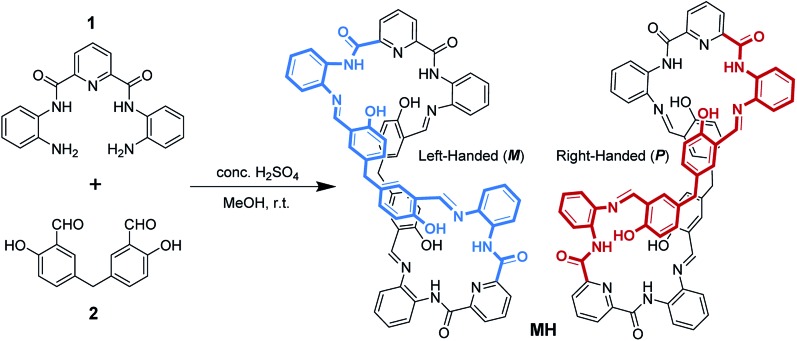
Synthetic route to the twisted macrocyclic host.

In earlier work, Sessler's group investigated in detail the effects of inorganic anions used as templates in the formation of 2,6-diamidopyridine pyrrolic macrocycles.^21^ Their results indicated that the choice of Brønsted acid is a critical factor defining the product distribution. As shown in Scheme 1, the Schiff base condensation reaction of *N*,*N*′-(6-amino-2-pyridyl)-1,3-dicarboximide (**1**) with 5,5′-methylene-bis-salicylaldehyde (**2**) in the presence of a Brønsted acid catalyst in methanol was first evaluated. In the case of the use of acid catalysts such as HCl, CH_3_COOH, CF_3_COOH, and HNO_3_, no precipitation was observed in the reaction, and multiple components that were produced were monitored by ^1^H NMR spectrometry. Concentrated H_2_SO_4_ was used as the catalyst to produce a large amount of light yellow precipitate from the reaction solution (ESI video†). Subsequently, a pure product with high yield (83%) was obtained just by using methanol to wash the precipitates several times. ^1^H NMR and MS analyses suggest that these precipitates are the [2 + 2] macrocycle **MH** (Fig. S1 and S2†).

**Fig. 1:**
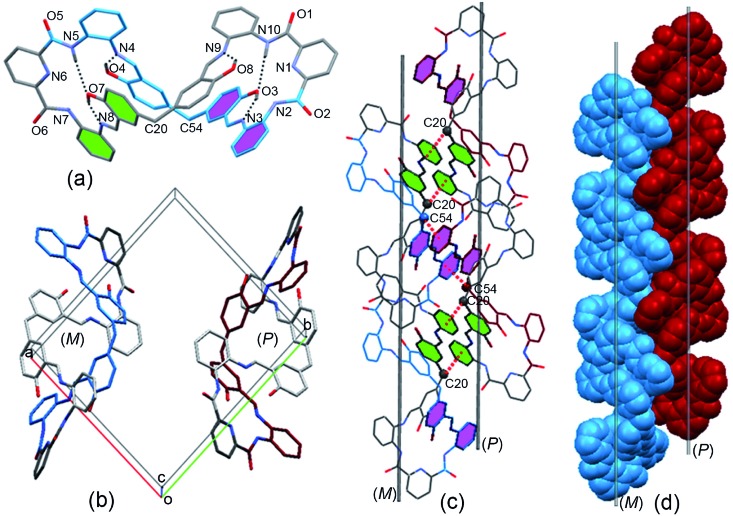
(a) X-ray crystal structure of the **MH** in left-handed helical conformation (*M*), (b) *M*- and *P*-helical structure of **MH** in the unit cell; (c) C(20)H···π(Cg1)^#1^ and C(54)H···π(Cg2)^#2^ interactions between the sp^3^ (CH) and the phenyl ring in the enantiomers of **MH**; Cg1: C28, C29, C30, C31, C32, C33; Cg2: C62, C63, C64, C65, C66, C67; #1: 2 – *x*, 1 – *y*, –*z*.; #2: 2 – *x*, –*y*, –*z*. (d) 1D columnar structure assembled from *M* and *P* helical assemblies. Hydrogen atoms and solvent molecules are omitted for clarity.

Crystals of **MH** were obtained from the solvent mixtures (ethyl acetate/dichloromethane, 1 : 1, v/v). Single-crystal X-ray diffraction provided unequivocal proof for the proposed structure and revealed that the macrocycle adopted a twisted three-dimensional (3D) helical structure in the solid state (Fig. 1a). Both helical conformations (left- and right-handed) were present in the unit cell (Fig. 1b). The 2,6-diamidopyridine fragments were in a *trans* orientation, and the amide oxygen atoms O1, O2, O5, and O6 pointed toward the exterior of the cavity. In each of the two bis(2-aminophenyl)pyridine-2,6-dicarboxamide units, the dihedral angles were 155° and 149° between the central pyridine ring and the two benzene rings located on the two “arms” (Fig. S3†). The dihedral angle between the two central pyridine rings (top) was 123.64° (Fig. S4†). As a result, the macrocyclic compound **MH** was twisted into a 3D helical conformation. The centroid–centroid distances between the two pairs of phenol rings located on the different side chains of the **MH** were 4.49 and 4.99 Å, respectively (Fig. S5†). Most interestingly, both of the two phenol rings were twisted in methylene-bridged fragments, in contrast to the free 5,5′-methylene-bis-salicylaldehyde groups (86.68°). The dihedral angles of the methylene linked phenol rings were determined to be 88.15° and 86.96°, respectively (Fig. S6†). From a structural viewpoint, such twisting behaviors induced by the single bond rotation of the bridged methylene played a crucial role in the helical tendency of the **MH**.

In addition, the 3D conformation of the **MH** was stabilized by multiple intermolecular hydrogen bonding interactions. For instance, as shown in Fig. 1a, protons of some of the phenol hydroxyl groups (O4, O8) were strongly hydrogen bonded to the Schiff base nitrogen atoms (N4, N9) receptively, while the other phenol hydroxyl moieties (O7, O3) were strongly hydrogen bonded to the imine (N8, N3) and amide (N5, N10) of the **MH***via* a pair of hydrogen bonds, in which the phenol hydroxyl group acted as a hydrogen bond donor and acceptor, with N···O distances ranging from 2.55 to 2.61 Å. Most importantly, it should be noted that a coplanar arrangement between the aromatic phenyl and phenol groups was observed on both side chains of the twisted **MH** (marked with green and violet color, respectively, Fig. 1a), and the dihedral angle between the two coplanar groups was determined to be 84.6° (Fig. S7†). As a result, such novel π-conjugated coplanar moieties of **MH** provide an ideal scaffold for the C–H···π interaction, resulting in 1D columnar self-assembly of the enantiomeric forms of **MH** (Fig. S8†).

As indicated in Fig. 1c, each right-handed helical (*P*) and left-handed helical (*M*) macrocyclic molecule was linked alternately to form a twisted 1D zigzag chain through intermolecular CH···π interactions between the bridge methylene (sp^3^-carbon C20 and C54) protons and the phenyl rings. The distances for CH···center of π interactions were 2.74 and 2.84 Å respectively. When viewed along the crystallographic *a* axis, the frameworks had a single (*P*) helix (red) and a single (*M*) helix (blue). The two helical chains packed parallel face to face gave a racemic mixture of two enantiomeric helices (Fig. 1d) and displayed 1D columnar structures (Fig. S9†). So far, reports on the assembly of columnar structures from macrocyclic compounds *via* CH···π interactions have been scarce.^22^,^23^ From observations of the X-ray solid structures of **MH**, it became clear that the methylene linker in the macrocycle played essential roles in defining the handed helical shapes of macrocyclic frameworks and in determining the assembly of 1D columnar structures. In spite of considerable progress in the design of metal-directed helicates by spontaneous self-assembly of conformationally flexible achiral ligands in recent years,^24^–^26^ there are few examples of the supramolecuar architecture from spontaneous self-assembly of pure organic *P* and *M* enantiomorphic macrocycle molecules, especially those derived from achiral precursors.

**Fig. 2:**
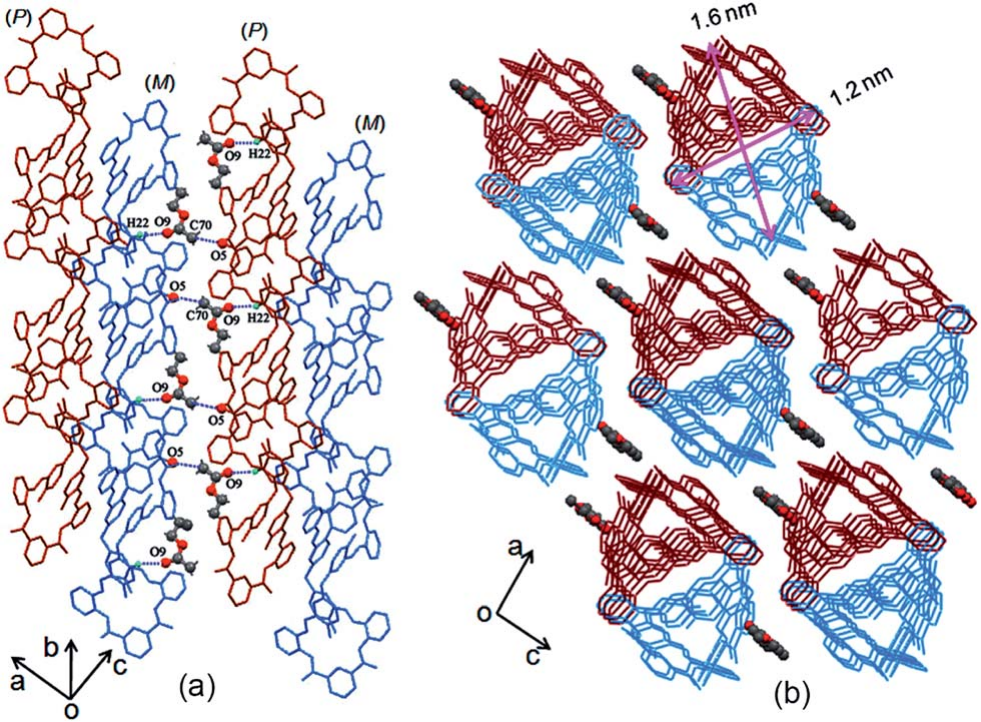
(a) Side and (b) top views of solvent molecules linked to 1D columns of **MH** in the crystal lattice.

The two adjacent organic 1D columnar structures are interconnected *via* intermolecular C–H···O hydrogen bonds between the amide oxygen (O5), phenol ring carbon (C22) protons in the **MH** molecules, and solvent molecules (CH_3_COOC_2_H_5_) (Fig. 2a). When viewed down the *b* axis, the individual 1D columnar structures in each case linked in a manner similar to that for adjacent pillars, forming 2D and 3D networks. The channels alongside these helices were filled with C–H···O hydrogen-bonded CH_3_COOC_2_H_5_ molecules, (Fig. 2b), and the individual 1D pillar displayed an oval section with dimensions of approximately 1.6 × 1.2 nm.

Generally, most of the supramolecular nanostructures have been constructed from macrocycles by their amphiphiles in solutions.^27^ Recently, Tominaga^28^–^30^ and Zheng^31^,^32^ revealed that organic particles could be formed from the spatial 3D geometry of nonamphiphilic macrocycles. Unlike the particles from the above-mentioned macrocycles, our result in this work revealed that the C–H···π interactions between the enantiomeric forms triggered core–shell-shaped microspheres, which have never been observed in organic macrocycle assembly and could be directly precipitated from the reaction solution with high yield. To the best of our knowledge, this is the first example of micro/nanoparticles from spontaneous self-assembly of pure organic *P* and *M* enantiomorphic twisted macrocyclic molecules from simple achiral starting materials *via* a one-step reaction.

**Fig. 3:**
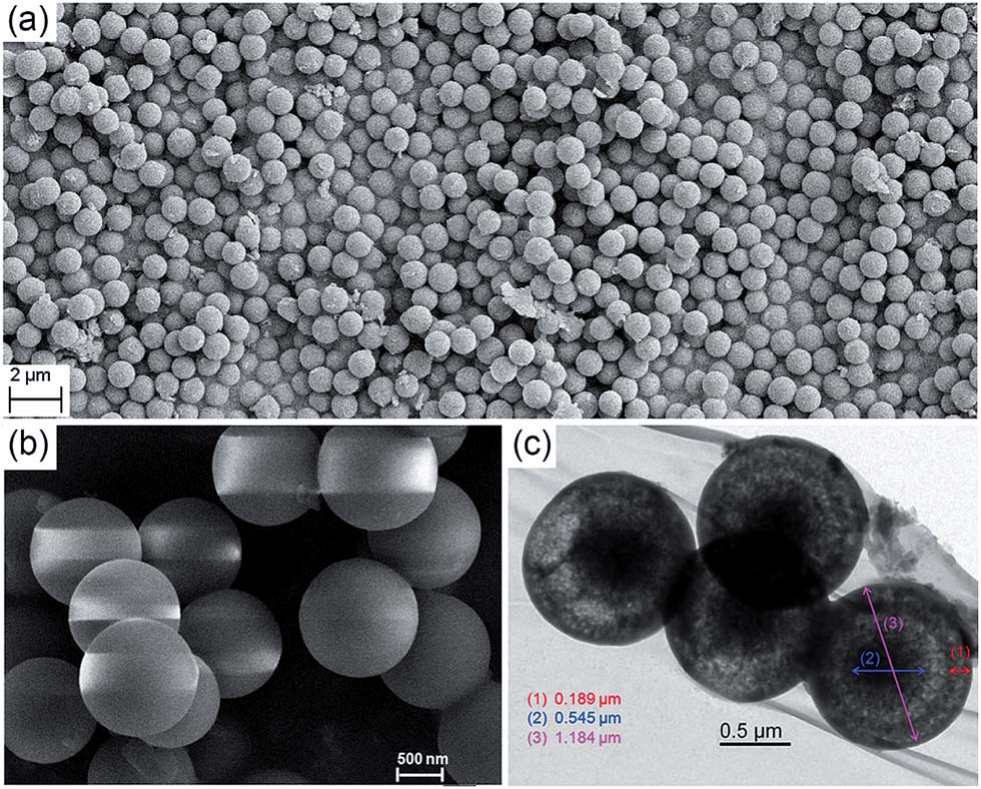
(a and b) SEM images of microspheres of **MH** at different magnifications; (c) TEM images of microspheres of **MH**.

Scanning electron microscopy (SEM) revealed that the precipitates were composed of microspheres with remarkable size uniformity, and that the contact edge between adjacent balls was clearly visible (Fig. 3a and b). The average size of the particles was found to be 1.2 μm. To determine whether the microspheres were solid balls or vesicles, methanol-dispersed microspheres were subjected to transmission electron microscopy (TEM). Surprisingly, it can be seen that the particles had a core in the hollow interiors, indicating that they were core–shell capsules. Fig. 3c suggests that the microspheres had a homogeneous core–shell morphology; the core surface and inner surface of the shell were also clearly visible (the thickness of the core and the shell is 0.54 ± 0.01 μm and 0.19 ± 0.01 μm, respectively). The diameter of the imaged microsphere was determined to be 1.18 ± 0.03 μm, which was in line with the particles observed by SEM.

Notably, differential scanning calorimetry and thermogravimetry indicated that the pure organic core–shell microspheres displayed high thermal stability (Fig. S10†). In an effort to gain detailed information on these microspheres' high-temperature resistance, SEM was performed. As shown in Fig. S11,† the microspheres, which were heated at different temperatures, exhibited high stability; no change was observed in terms of their size and shape after they were heated at 100, 200, and 225 °C. The particles began to fuse with each other as the temperature rose to 230 °C, and finally merged into a block morphology at 232 °C.

As mentioned previously, the core–shell particles were obtained directly from the precipitation from the Schiff base reaction. Actually, the precipitates formed fast in the organic reaction. As shown in a video (ESI†), which contains substrates **1** and **2**, a light yellow precipitate almost immediately appeared as the reaction was stirred at room temperature upon addition of catalytic equivalents of sulfuric acid to the methanol solution. Therefore, the simple and facile fabrication conditions of the particles allowed realization of the construction of core–shell assemblies from the enantiomeric macrocyclic molecules. Direct collection of the precipitates from the solution may be done by controlling the reaction time.

**Fig. 4:**
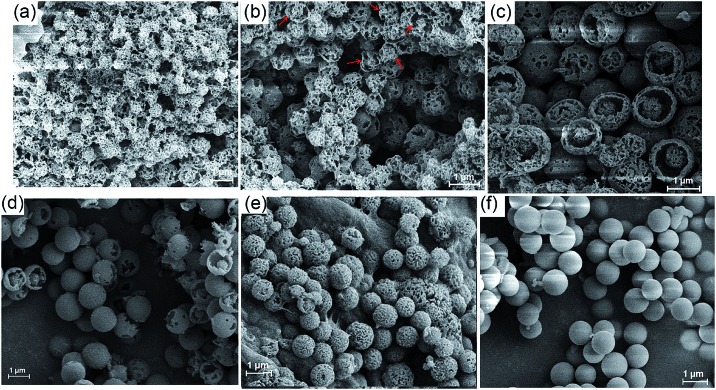
SEM images of the precipitates of **MH** from methanol reaction solution at different times: (a) 1 min, (b) 3 min, (c) 5 min, (d) 7 min, (e) 10 min, and (f) 1 h.

Initially, when the reaction time was controlled within 2 min, the resulting precipitates were collected and subjected to SEM analysis. As shown in Fig. 4a and S12,† a varying honeycomb-like morphology was observed, and no ball or other particle shape was found in the image at first glance. In contrast, a tendency towards the formation of porous spheres could be discerned with closer inspection. The spherical shape became clear and accompanied a core–shell morphology when the reaction time was controlled within 4 min (Fig. 4b and S13†). The morphology was different from that previously described when the reaction time was increased to 6 min. As seen in Fig. 4c and S14,† a large number of perfect porous microspheres with a rough surface were obviously formed, especially some core–shell microspheres resembling broken eggs, in which the core or the “yolk” was perfect. As the reaction time was fixed to 8 min (Fig. 4d and S15†), no changes in the size and shape of the particles occurred, but the bowl-shaped shell showed a remarkable tendency to assemble into a ball, generating the microporous sphere. Fig. 4e and S16–18† show the stage in which the perfect microspheres formed within 15 min. The porous surface became smooth as the reaction time continued to increase to 1 h (Fig. 4f, S19 and S20†). In addition, the ^1^H NMR spectra of the precipitates which were produced at different reaction times displayed similar proton peaks with **MH** (Fig. S21†), indicating that all of the solid samples were mainly composed of the **MH**. Consequently, these results provide solid proof for the formation of core–shell-shaped microspheres by spontaneous self-assembly of the twisted enantiomers of **MH**, which may be the result of the thermodynamic and kinetic balance in the Schiff base reaction procedure.

**Scheme 2:**
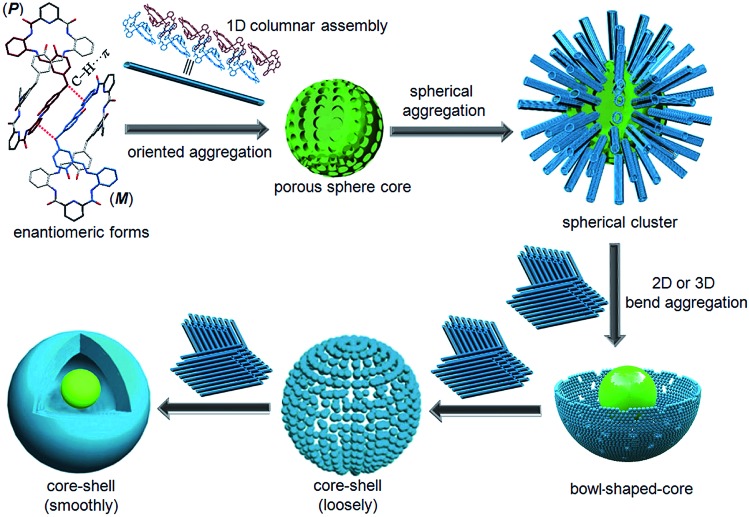
Proposed growth process of the core–shell-based microsphere of **MH**.

Combining this issue and the observations shown above, we can schematically illustrate the proposed model for the formation of the enantiomeric forms of the **MH**-based core–shell architecture in Scheme 2. It includes the formation of a 1D column by the CH···π interactions between the enantiomeric conformers of **MH** and the solvent-linked 2D and 3D alignments of the 1D columns. In supramolecular chemistry, the general formation mechanism of noncovalent interactions triggered by organic particles has been proposed. This is similar to the formation model of lipid spheres, in which the 2D planar bilayers are assumed to be involved.^33^ Under certain conditions, the planar bilayers are more favorable for closed bilayers rather than infinite planar bilayers. This is because the energetically unfavorable edges in a closed bilayer, which is also entropically favored, are eliminated at a finite aggregation number.^34^,^35^ As indicated by the X-ray structure of **MH** in the solid state, the CH···π interactions between the enantiomers of **MH** in this work play a key role in the formation of 1D columns, which further makes the 1D columnar assembly comprise oriented aggregates. Solvent molecules such as methanol then link the oriented 1D columns to form the 2D or 3D aggregates *via* hydrogen bonds (evidence from hydrogen bonds between the solvent of CH_3_COOC_2_H_5_ and the 1D enantiomeric forms assembled in the solid state as shown in Fig. 2). The mechanism can be described in detail as follows. (1) At the early stage of the Schiff base reaction, the 1D column oriented assembly, the main component with a large amount of **MH**, is generated. SEM indicates that the sphere core is initially formed at this stage (Fig. 4a). (2) A 2D or 3D aggregate fragment with a certain size is linked by the solvent molecules and starts to bend to reduce its total energy. SEM suggests that the assembly of the spherical cluster core is almost complete, and a bowl-shaped core sphere generally formed (Fig. 4b–d). (3) The organic reaction is finished and the solvent linked curved aggregate fragments, which generates a loosely microporous sphere (Fig. 4e). (4) Some of the remaining aggregate fragments of **MH** are further linked by the solvent molecules into the porous shell to produce smooth microsphere particles (Fig. 4f). Consequently, it is believed that the shape including the core of the microsphere is dependent on the enantiomeric triggered 1D columnar assembly. The hydrogen bonding between the solvents (such as methanol) and the 1D columnar structure plays a crucial role in determining the radius of the microspheres.

**Fig. 5:**
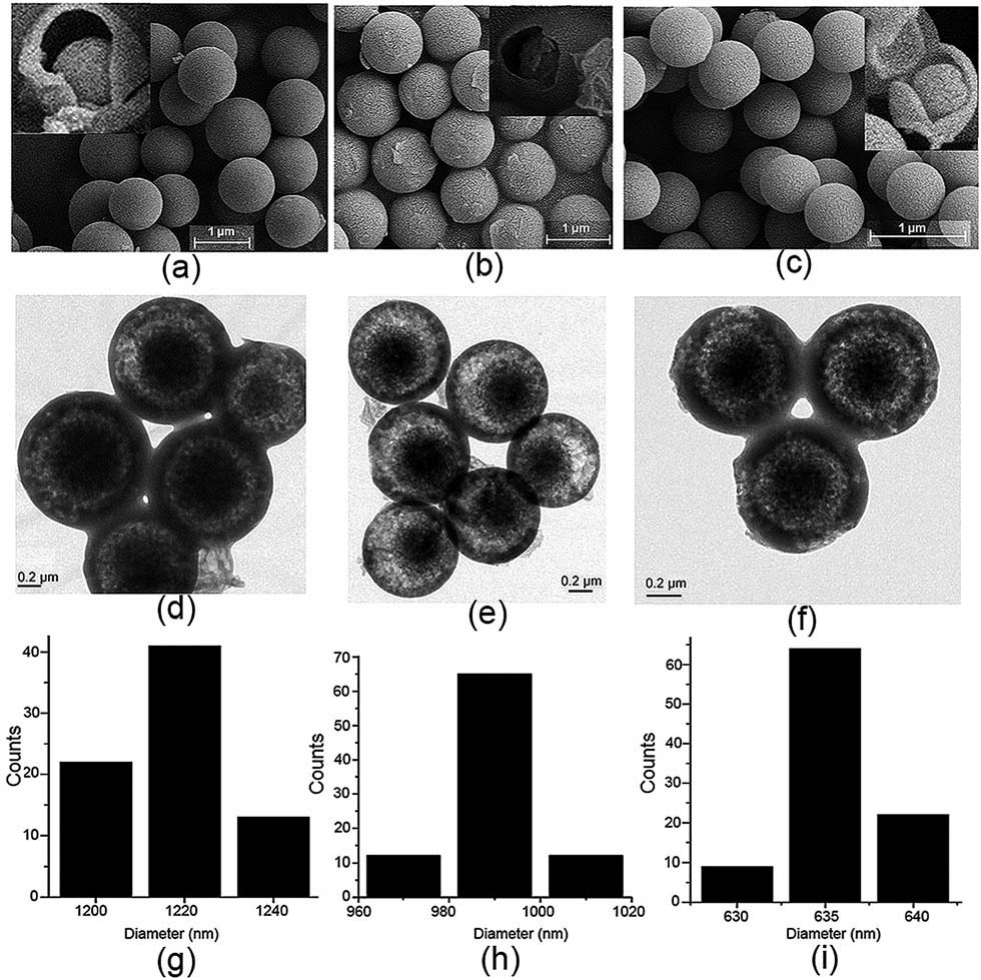
(a–c) SEM images (inset: the related precipitates after heating at 210 °C for 5 min), (d–f) TEM images, and (g–i) particle size distribution diagram of the formed microspheres of **MH** in the case of addition of 0, 1.0, and 3.0 mL of water to the reaction solution.

For a better understanding of the proposed mechanism of the formation of the core–shell based microsphere, water, a more strongly hydrogen-bonding molecule, was added to the reaction solution under the same conditions and the reaction was carried out for 3 h. As clearly shown by SEM and TEM in Fig. S22–S26,† the size of the microsphere was dramatically decreased with increasing volume of the water, while no change was observed in the core–shell architecture and the shape of the microsphere. In particular, the diameter of the core–shell-based microsphere could be regularly controlled at 1.22 ± 0.03 μm, 990 ± 20 nm, and 635 ± 5 nm in the case of addition of 0, 1.0, and 3.0 mL of water to 20, 19, and 17 mL of methanol solution, respectively (Fig. 5). No change was noted for the size and the shape of the microsphere when more water was added to the reactions solution. ^1^H NMR spectra suggested that such different size appended spherical particles were fabricated from the assemblies of the **MH** molecules (Fig. S27†). Obviously, the decreased size of the microsphere can probably be ascribed to the stronger hydrogen bonding between the water molecules and the 1D columns based on enantiomeric conformers compared to methanol molecules, which thus led to the formation of compact 2D or 3D aggregates for the microsphere. Essentially, these observations fully support and are in line with the proposed mechanism for the formation of the core–shell-shaped architecture.

## Conclusions

In summary, we have established a novel and facile method for the fabrication of pure homogeneous and highly thermostable organic nanoparticles with core–shell architecture. The synthesis was done *via* a one-step simple organic reaction at room temperature with a high yield. This approach greatly decreased the number of tedious synthetic steps and produced a system with tunable size for organic micro/nanoparticles. The results reveal that the single-bond rotation of the bridged methylene on the bis-salicylaldehyde moiety is very important to formation of the twisted enantiomeric structure of **MH** from the achiral precursor by Schiff base reaction. Furthermore, the enantiomeric forms in the crystalline phase in equimolar proportions to form two racemic helix structures (1D column), which are complementary to each other and the orientation of which is stabilized by intermolecular C–H···π interactions. We propose that this plays a crucial role in the formation of the core–shell-based microsphere structure. The present work demonstrated a new phenomenon of organic particle growth, as well as providing a new viewpoint and application of enantiomeric structures. Further studies on the synthesis and properties of such twisted chiral macrocycles are currently being carried out.

## Experimental

### Methods

*N*,*N*'-(6-Amino-2-pyridyl)-1,3-dicarboximide (**1**)^36^ and 5,5′-methylene bis-salicylaldehyde (**2**)^37^ were synthesized according to methods described in the literature.

#### Synthesis of the macrocyclic host (**MH**) and organic particles

**1** (174.0 mg, 0.5 mmol) and **2** (128.0 mg, 0.5 mmol) were dissolved in methanol solution (30–40.0 mL) in a round-bottomed flask for 10 min, and conc. H_2_SO_4_ (20.0–30.0 μL) was added to the solution. The resulting mixture was stirred (800–1000 rpm) at room temperature (15–30 °C) for 3 h. Then, the reaction mixture was filtered to give the crude solid product, and the residue was washed with methanol three times to afford a light yellow solid compound **MH** (470 mg, 83%). SEM and TEM images confirmed that the yellow solid is the core–shell based microparticles (∼1.22 μm). Mp > 250 °C. ^1^H NMR (400 MHz, *d*_6_-DMSO) *δ* (ppm) 12.77 (s, 4H, OH), 10.36 (s, 4H, O=C–NH), 8.51 (d, *J* = 8 Hz, 2H, Py–H), 8.38 (t, *J* = 16 Hz, 2H, Py–H), 8.22 (d, *J* = 8 Hz, 4H, Ar–H), 8.04 (s, 4H, N=CH), 7.39 (t, 4H, Ar–H), 7.33 (s, 4H, Ar–H), 7.14 (t, 4H, Ar–H), 6.97 (d, *J* = 8 Hz, 4H, Ar–H), 6.87 (d, *J* = 8 Hz, 4H, Ar–H), 6.13 (d, *J* = 8 Hz, 4H, Ar–H), 3.58 (s, 4H, CH_2_). ^13^C NMR (100 MHz, CDCl_3_) *δ* 161.68, 159.27, 149.54, 139.19, 138.18, 133.68, 132.22, 131.41, 130.88, 128.65, 126.38, 125.54, 124.78, 118.96, 117.63, 115.92, 39.33, 29.33. ESI-MS (*m*/*z*): calcd for [M + H]^+^ [C_68_H_51_N_10_O_8_]^+^*m*/*z* = 1135.38; found *m*/*z* = 1135.3877.

#### X-ray structure of **MH**

Single crystals were obtained from a crystal grown by evaporation of **MH** (50.0 mg) in a solution mixture (20.0 mL, ethyl acetate/dichloromethane, 1 : 1, v/v). Crystal data for **MH**: (C_68_H_50_N_10_O_8_)·2CH_3_COOC_2_H_5_·CH_2_Cl_2_, *M*_r_ = 1394.30, triclinic, space group *P*1[combining macron], *a* = 13.861(5) Å, *b* = 15.962(6) Å, *c* = 17.258(6) Å, *α* = 69.871(6)°, *β* = 75.157(6)°, *γ* = 81.221(7)°, *V* = 3457(2) Å^3^, *Z* = 2, *D*_c_ = 1.340 g cm^–3^, *R*_1_ = 0.0749 (*I* > 2*σ*(*I*)), w*R*_2_ = 0.1296 (all data), GoF = 0.804. CCDC ; 1856480.

#### Water controlled organic particle size

**1** (87.0 mg, 0.225 mmol) and **2** (64.0 mg, 0.225 mmol) were dissolved in 20.0 mL solution mixture (CH_3_OH/H_2_O, 19 : 1, v/v, or CH_3_OH/H_2_O, 17 : 3, v/v) in a round-bottomed flask for 10 min, and conc. H_2_SO_4_ (10.0–15.0 μL) was added to the solution. The resulting mixture was stirred (800–1000 rpm) at room temperature (15–30 °C) for 3 h. Then, the reaction mixture was filtered to give the crude solid product, and the residue was washed with methanol three times to afford a light yellow solid compound **MH** (yield, 80–70%). SEM and TEM images confirmed that the yellow solid is the core–shell based microparticles (∼990 nm and ∼635 nm). No change was observed for the size and the shape of the microspheres when more water was added to the reaction solution.

## Conflicts of interest

The authors declare no competing financial interests.

## Supplementary Material

Supplementary information

Supplementary movie

Crystal structure data
