# Supplementary material for: Organic core–shell-shaped micro/nanoparticles from twisted macrocycles in Schiff base reaction
Source: Chem Sci. 2018 Oct 15;10(2):490–6. doi: 10.1039/c8sc03824d (PMC6354836; doi:10.1039/c8sc03824d)
Supplement: Supplementary file 1 [file SC-010-C8SC03824D-s001.pdf]

Electronic Supplementary Material (ESI) for Chemical Science.
This journal is © The Royal Society of Chemistry 2018

Supporting Information

Organic core–shell-shaped micro/nanoparticles from twisted macrocycles in Schiff base reaction

Huaiyu Chen, Chao Huang, Yazhou Ding, Qi-Long Zhang, Bi-Xue Zhu* and Xin-Long Ni*

Key Laboratory of Macrocyclic and Supramolecular Chemistry of Guizhou Province, Guizhou University, Guiyang, Guizhou 550025, China

E-mail: bxzhu@gzu.edu.cn (B.-X. Zhu)

E-mail: longni333@163.com (X.-L. Ni)

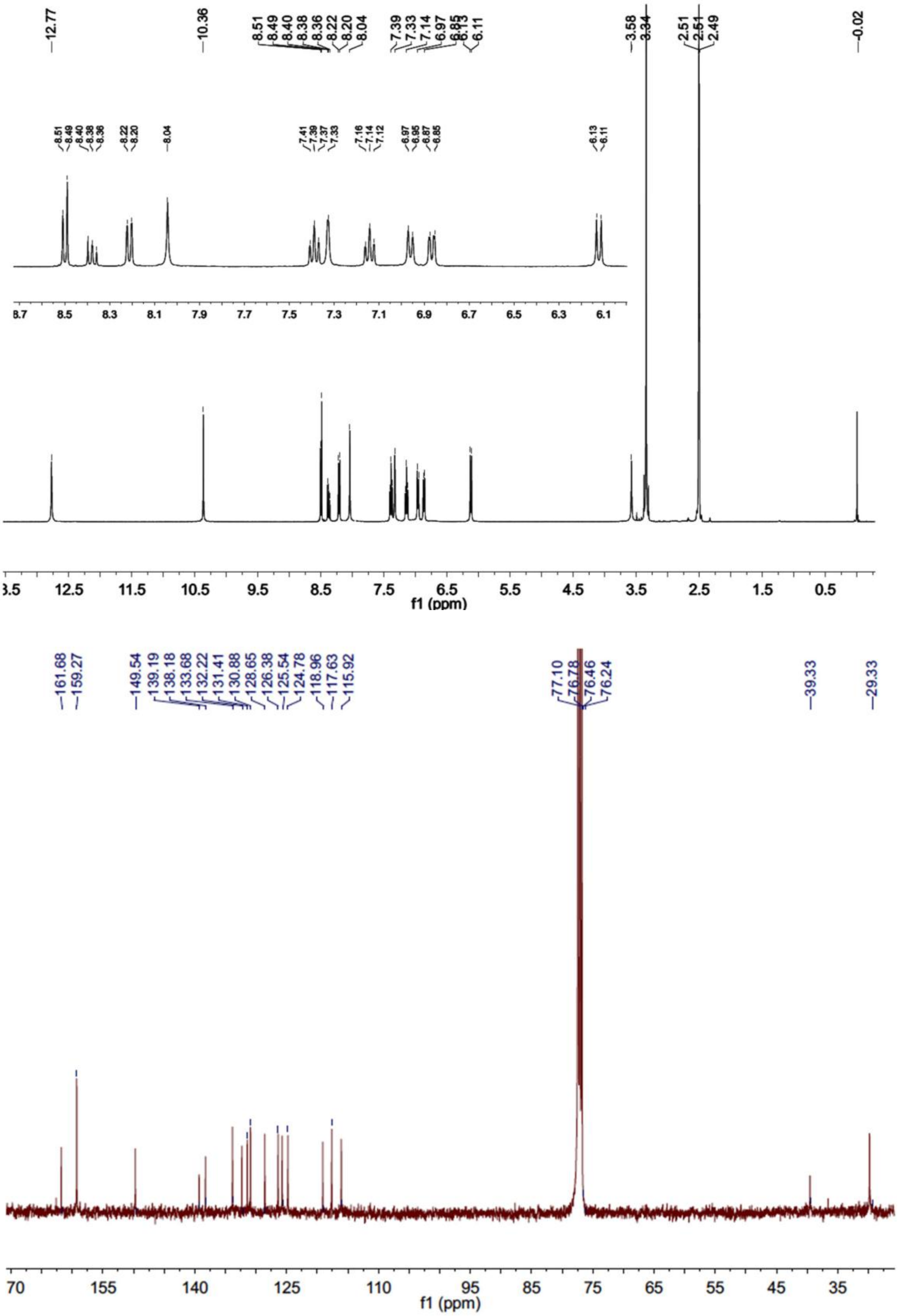

Figure S1. ^{1}H NMR spectra (400 MHz, d_6-DMSO) and ^{13}C NMR (100 MHz,$CDCl_3$) spectra of **MH**.

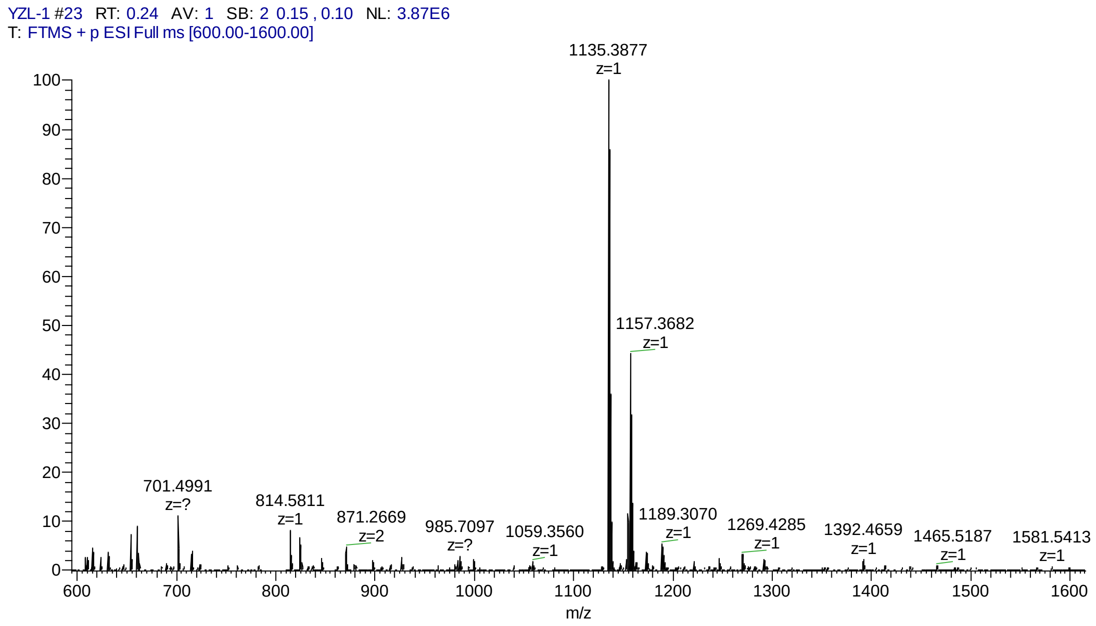

Figure S2. Mass spectra of **MH**. calcd for $[M + H]^+$ $[C_{68}H_{51}N_{10}O_8]^+$ *m*/*z* = 1135.38; found *m*/*z* = 1135.3877.

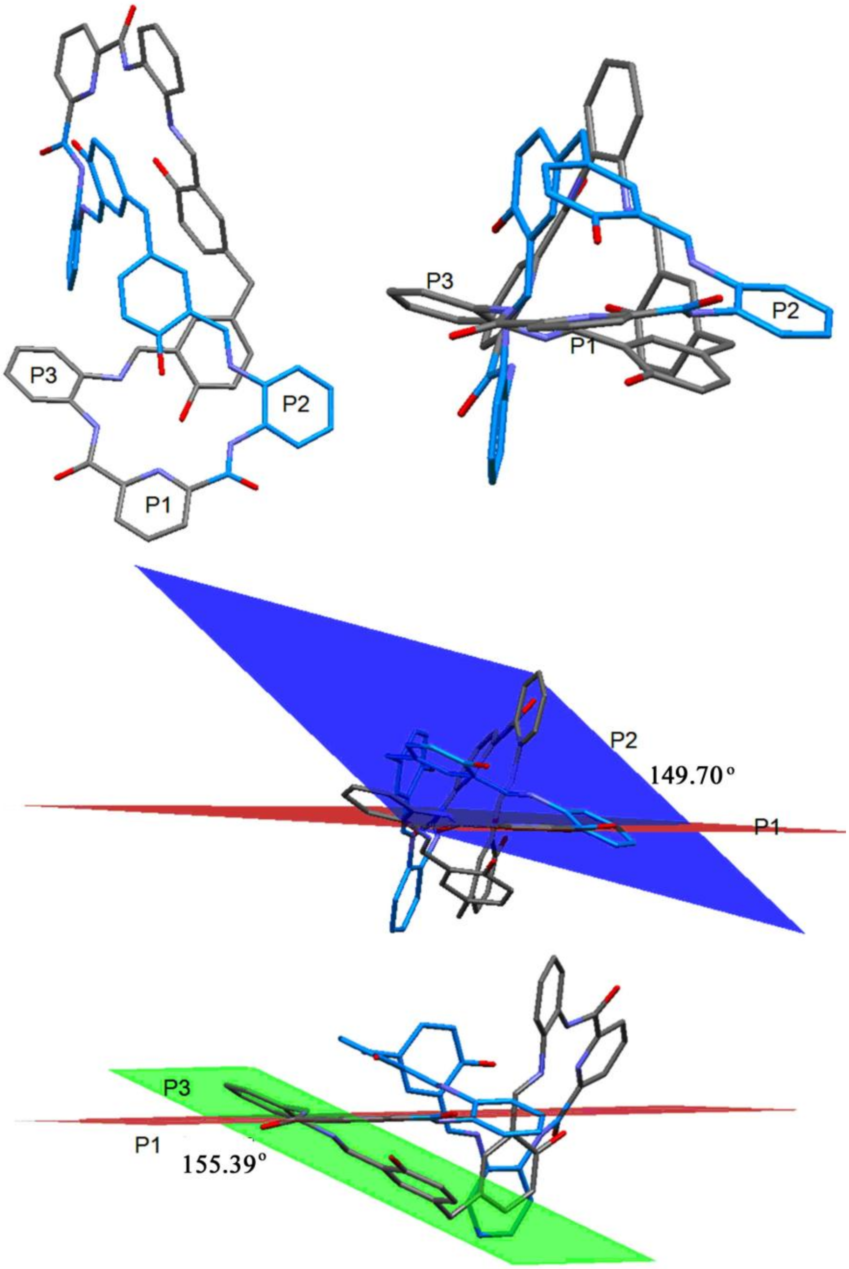

Figure S3. Dihedral angles between the two benzene rings (P2 and P3) and the central pyridine ring (P1), respectively.

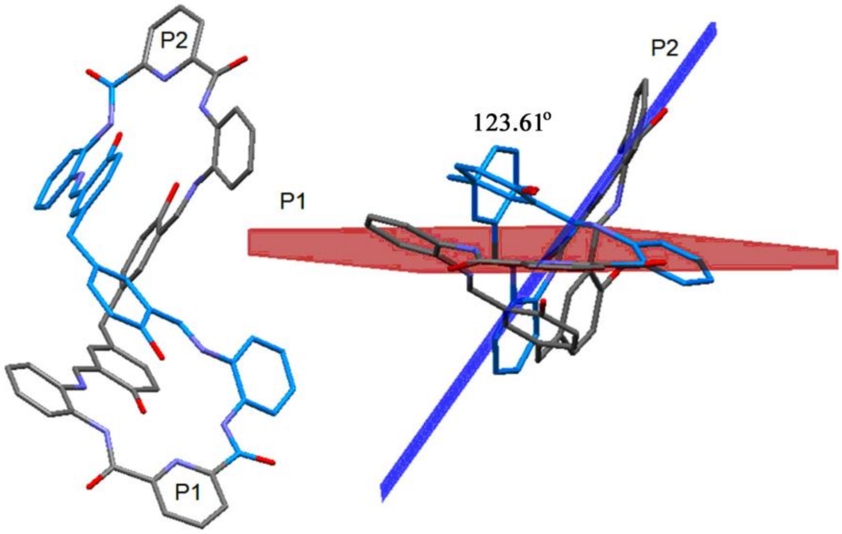

Figure S4. Dihedral angles between the central pyridine ring (P1 and P2).

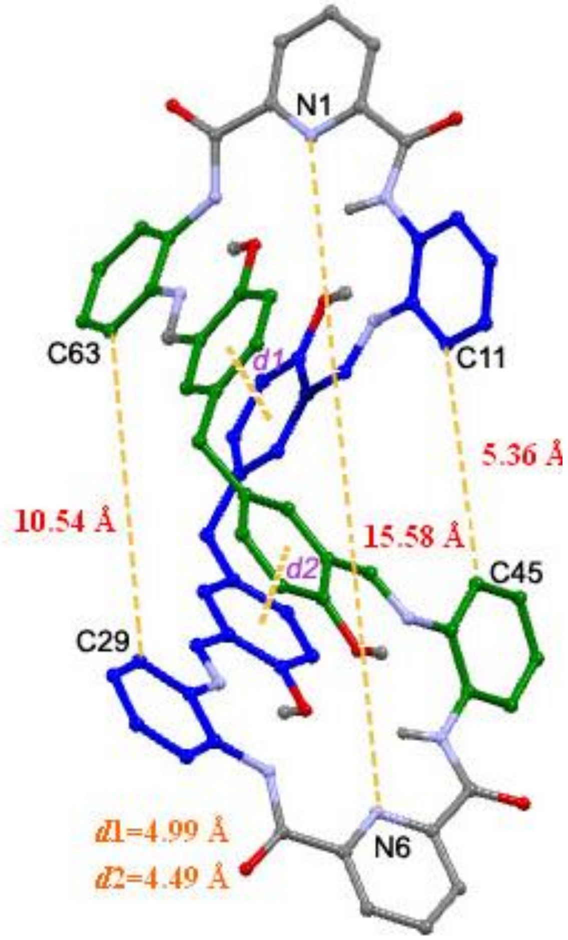

Figure S5. Distances between the different groups of **MH**.

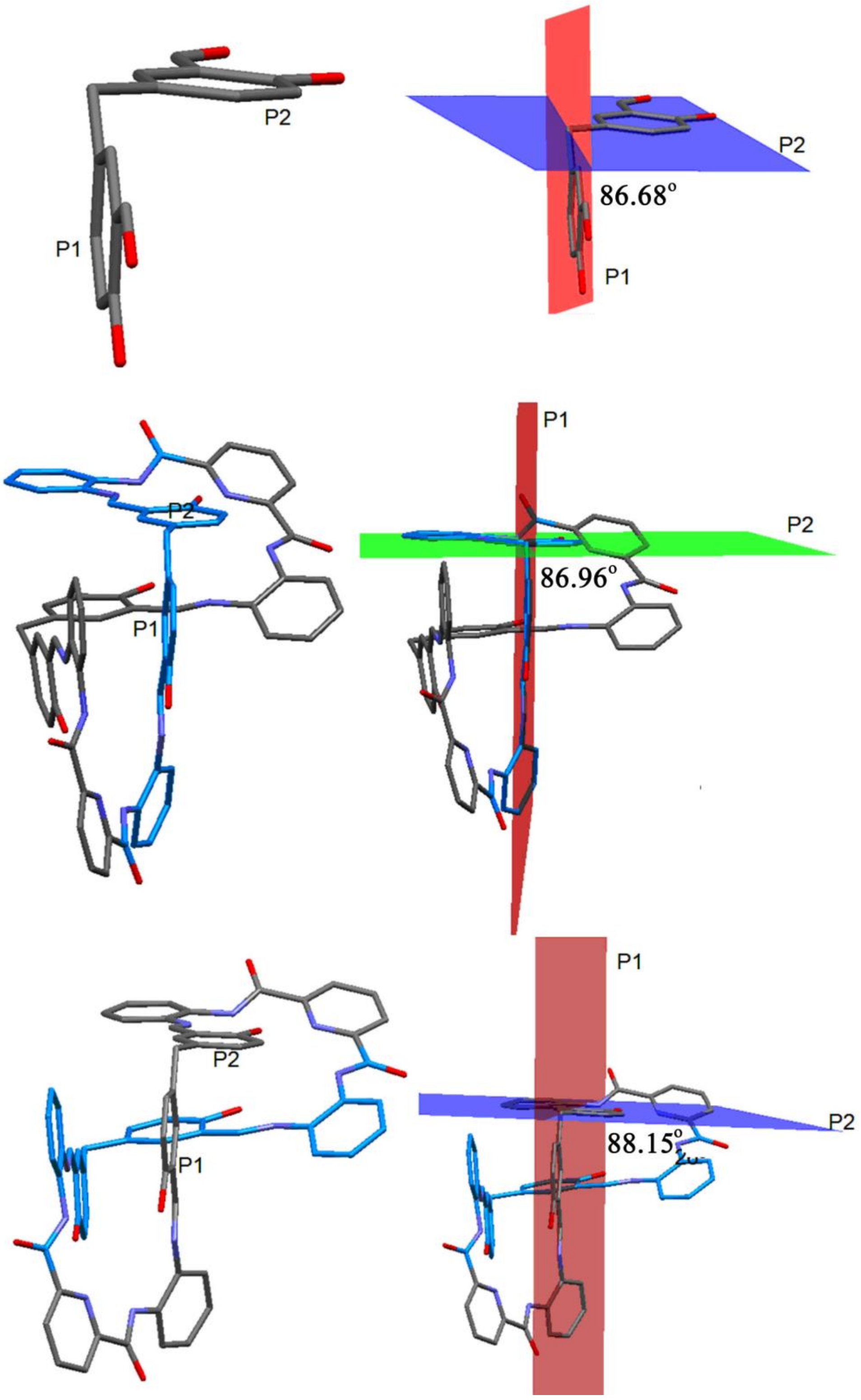

Figure S6. Dihedral angles between of the two phenol rings on the 5,5'-methylene bis-salicylaldehyde groups in free or fixed by the twisted **MH**, respectively.

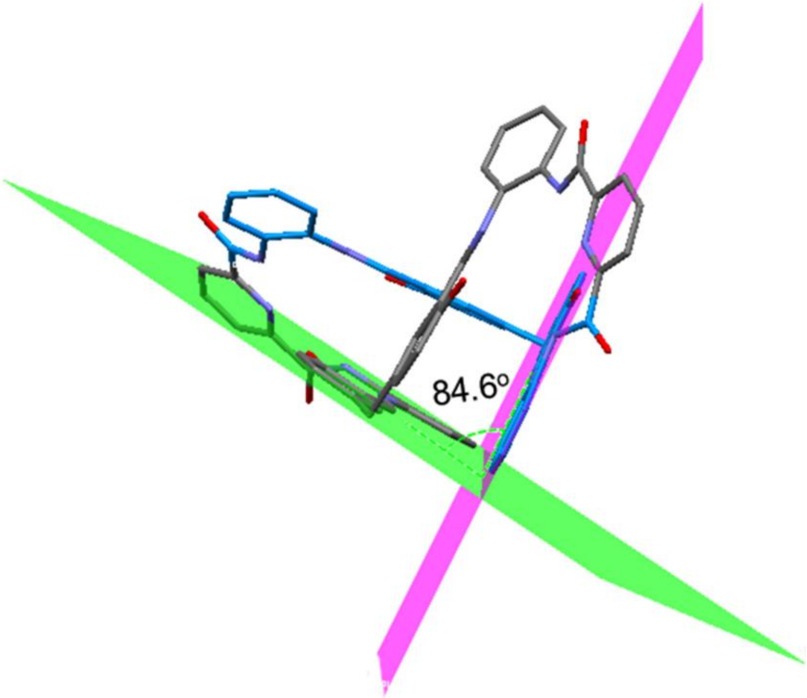

Figure S7. The dihedral angle between the two coplanar units was determined to be 84.6°.

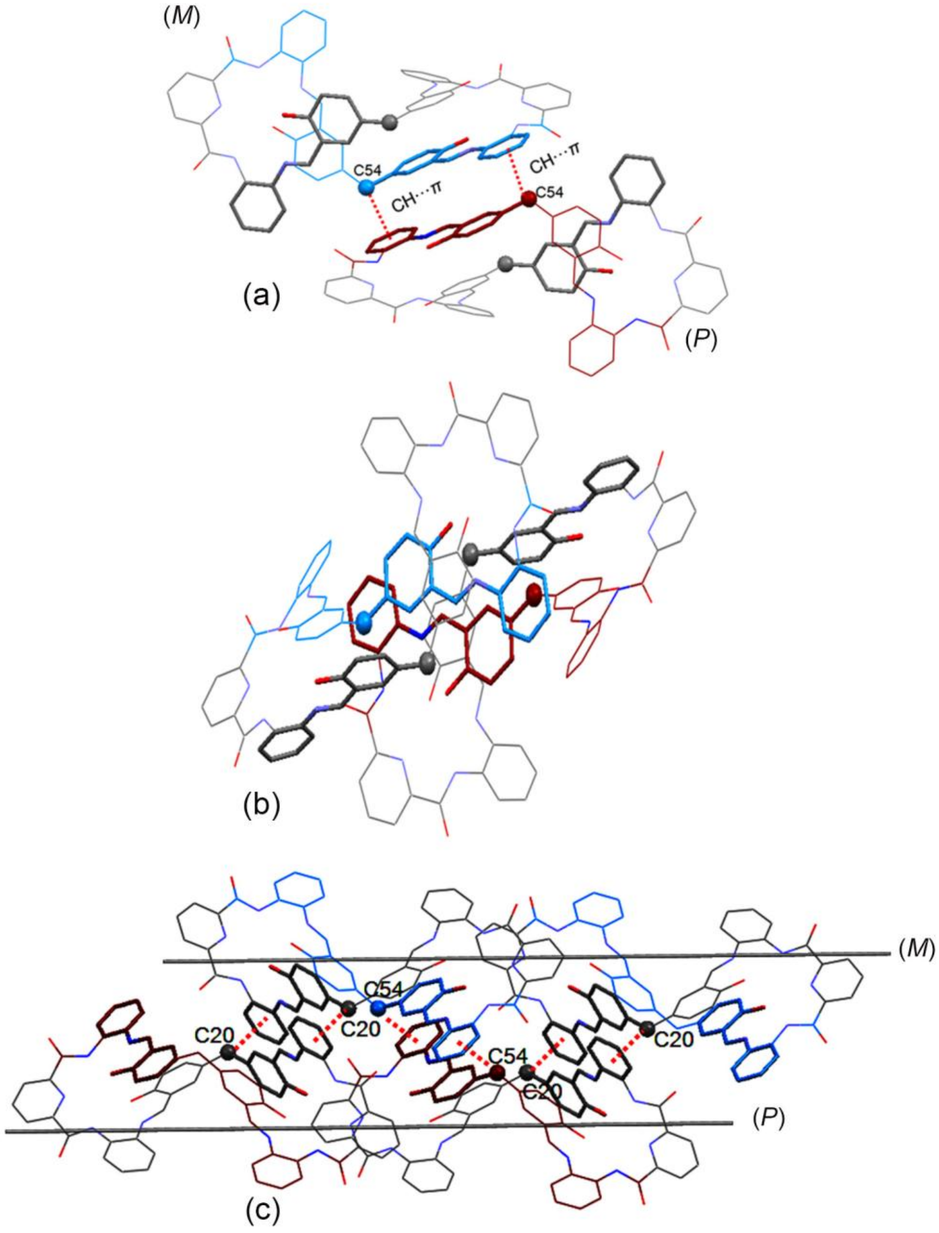

Figure S8. C−H···π interaction between the enantiomeric forms of MH (a) side view, (b) top view. (c) C−H···π interaction directed 1D columnar self-assembly of the enantiomeric forms of **MH**.

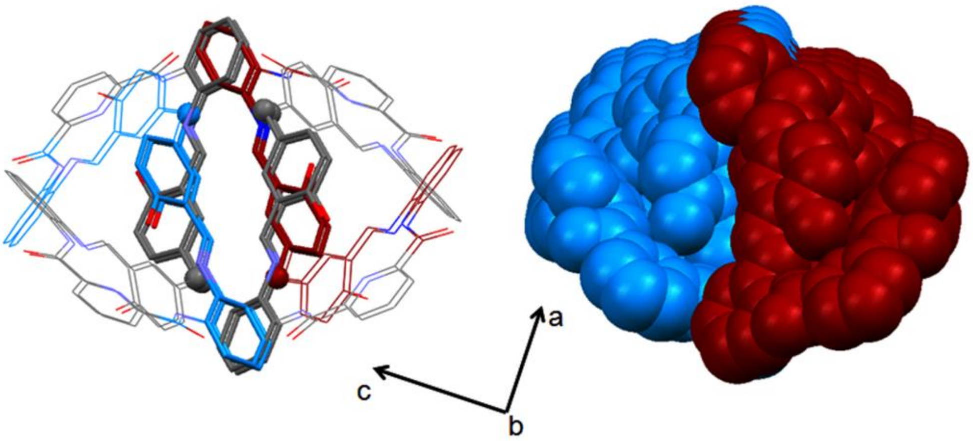

Figure S9. Top view of the 1D columnar structure from assembly of the enantiomeric forms of **MH**.

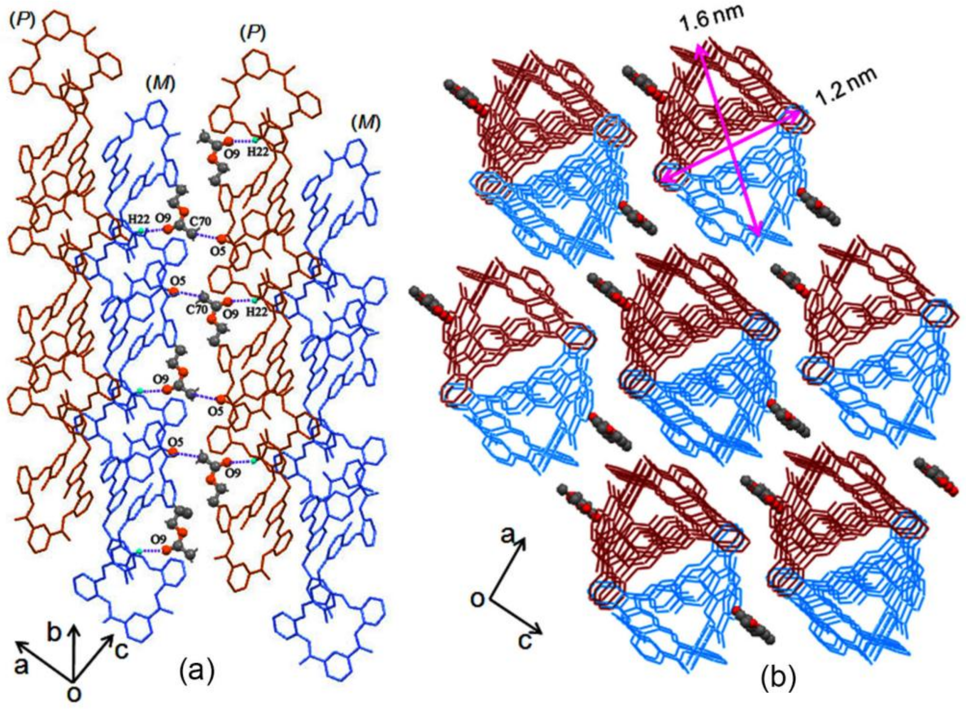

Figure S10. Solvent-linked supramolecular assembly of the enantiomeric forms of **MH**. (a) Side and (b) top views of solvent molecules linked to 1D columns of **MH** in the crystal lattice.

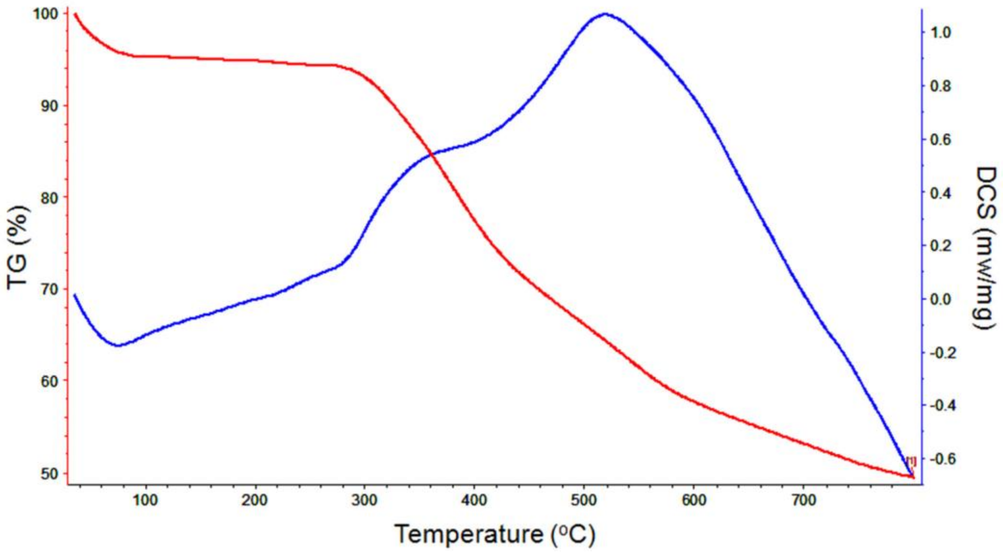

Figure S11. Thermogravimetric analysis diagram of compound **MH** under N_2 protect.

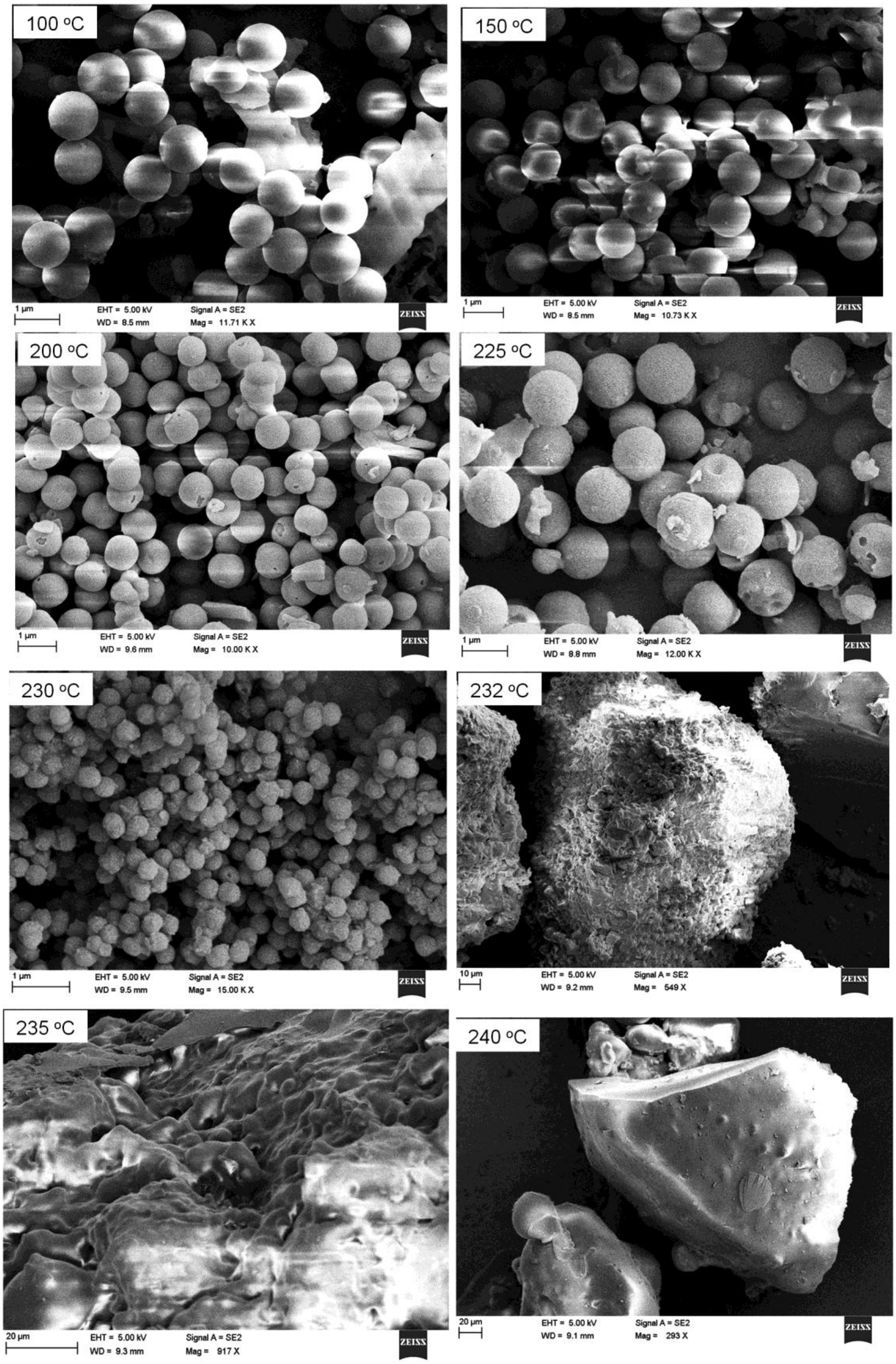

Figure S12. SEM images of **MH** was heated at different temperature (100 ^{o}C, 150 ^{o}C, 200 ^{o}C, 225 ^{o}C, 230 ^{o}C, 232 ^{o}C, 235 ^{o}C and 240 ^{o}C) under N_2 protection.

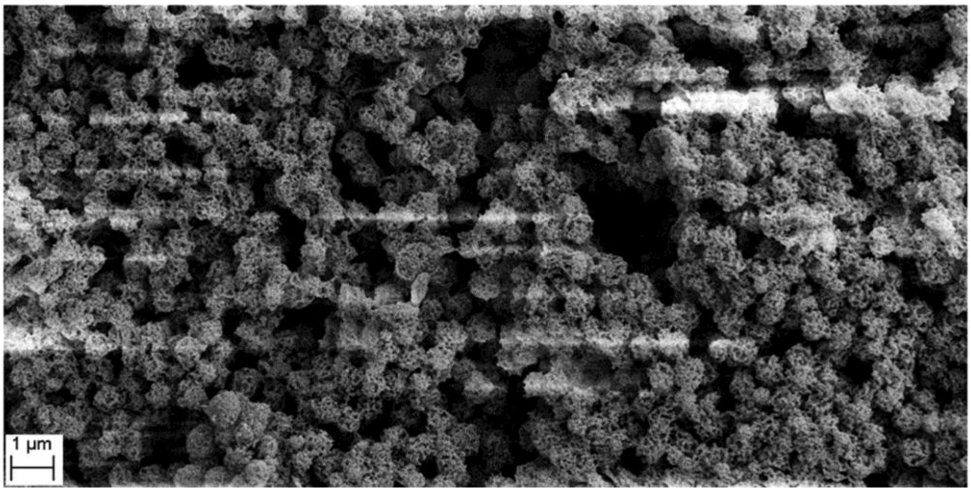

Figure S13. SEM images of the precipitate of **MH** at 2 min.

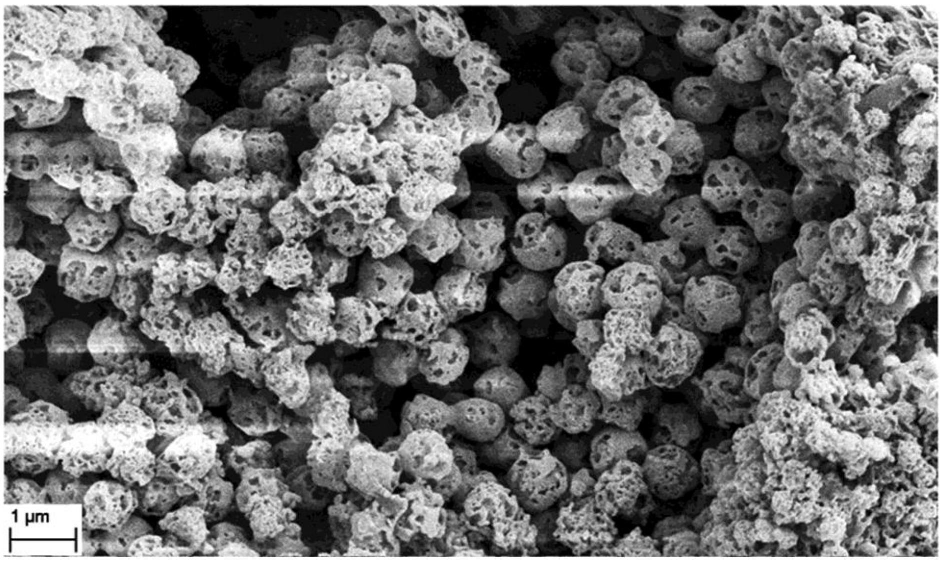

Figure S14. SEM images of the precipitate of **MH** at 4 min.

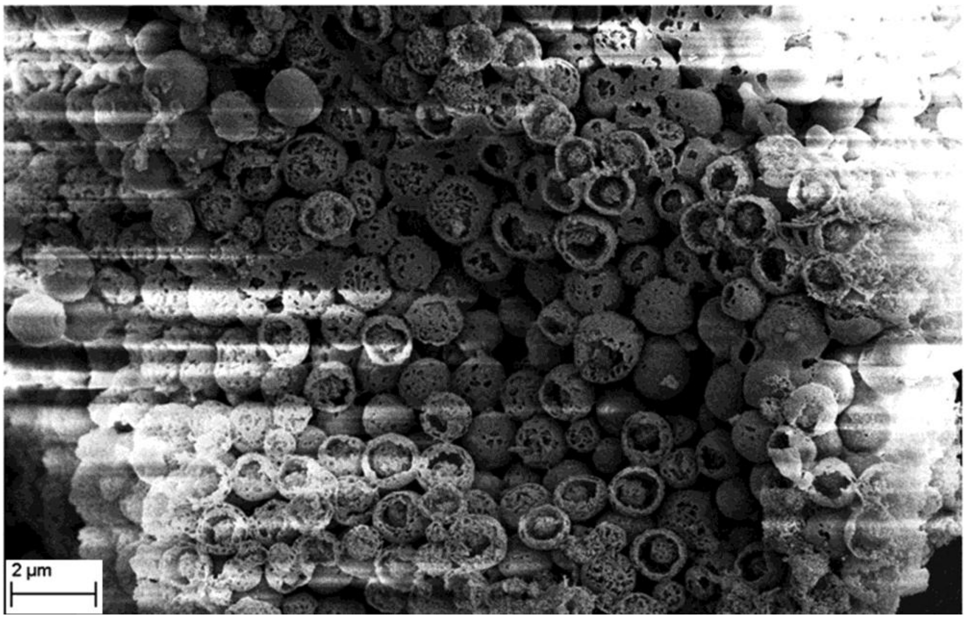

Figure S15. SEM images of the precipitate of **MH** at 6 min.

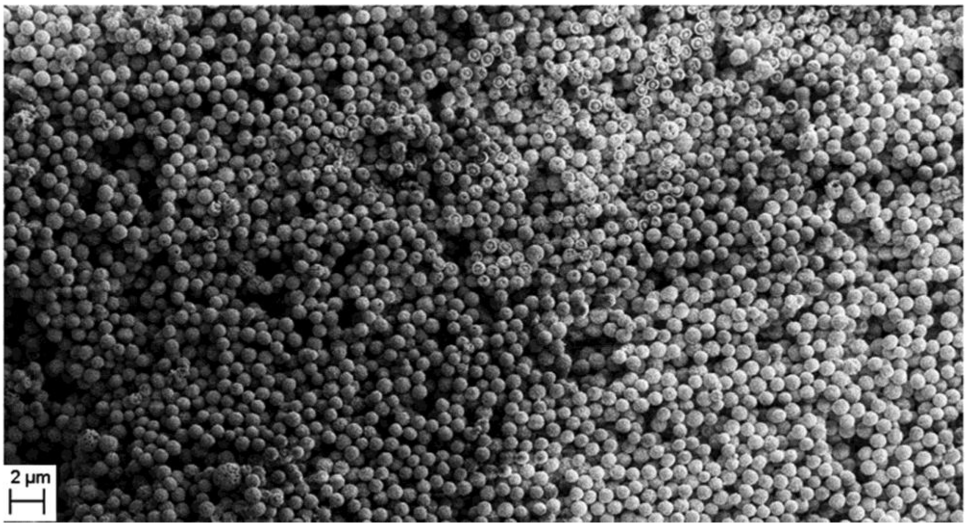

Figure S16. SEM images of the precipitate of **MH** at 8 min.

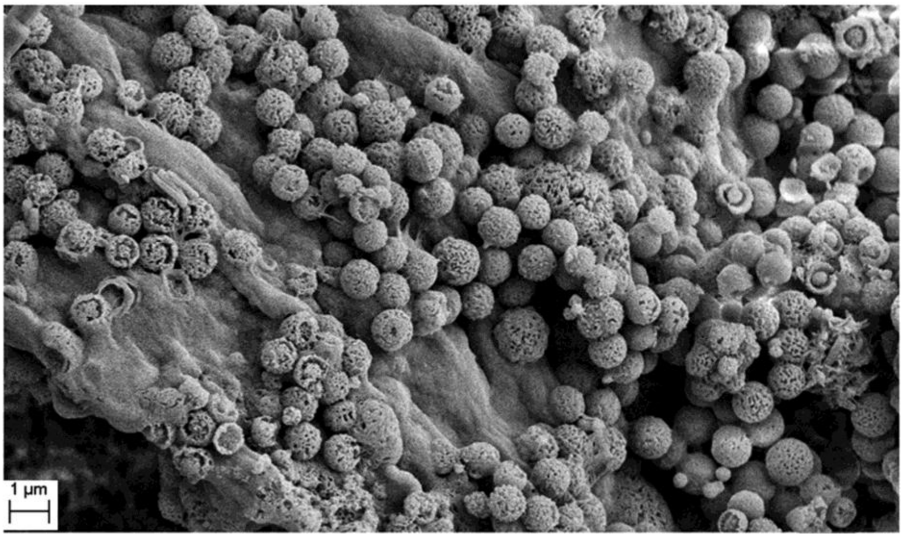

Figure S17. SEM images of the precipitate of **MH** at 10 min.

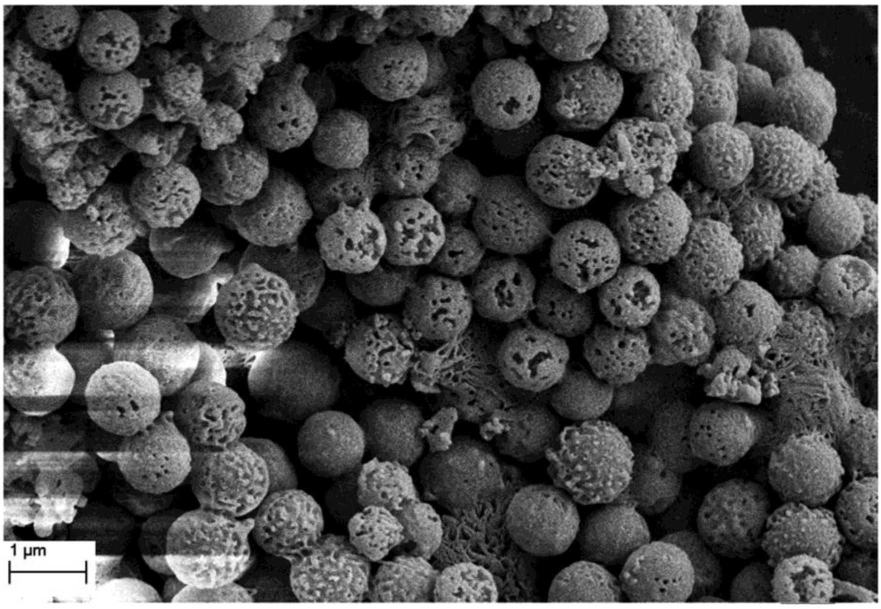

Figure S18. SEM images of the precipitate of **MH** at 12 min.

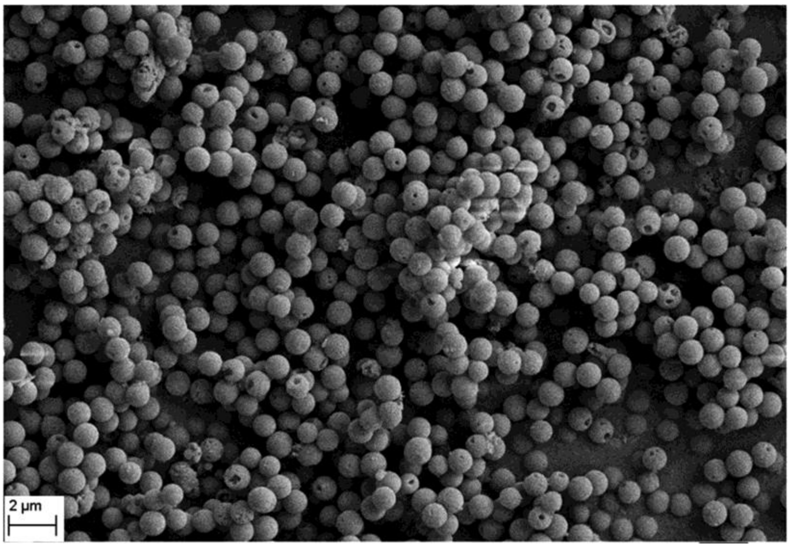

Figure S19. SEM images of the precipitate of **MH** at 15 min.

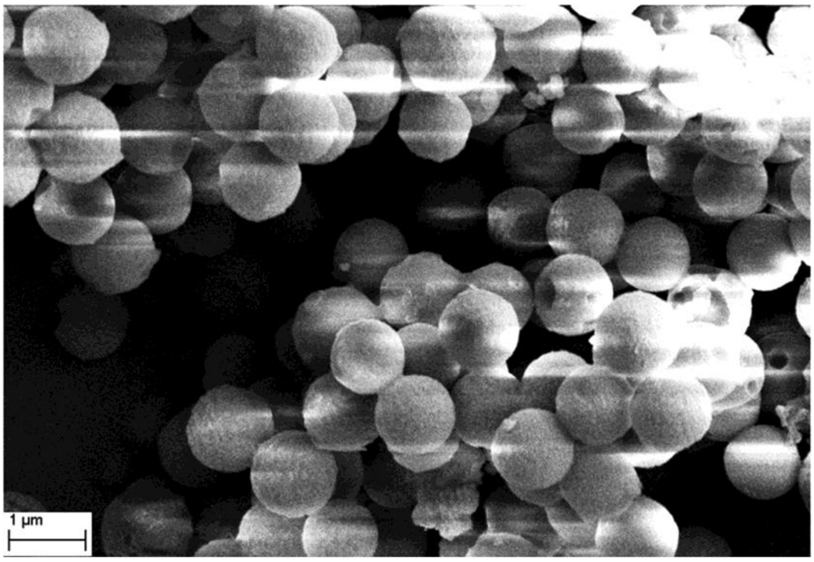

Figure S20. SEM images of the precipitate of **MH** at 30 min.

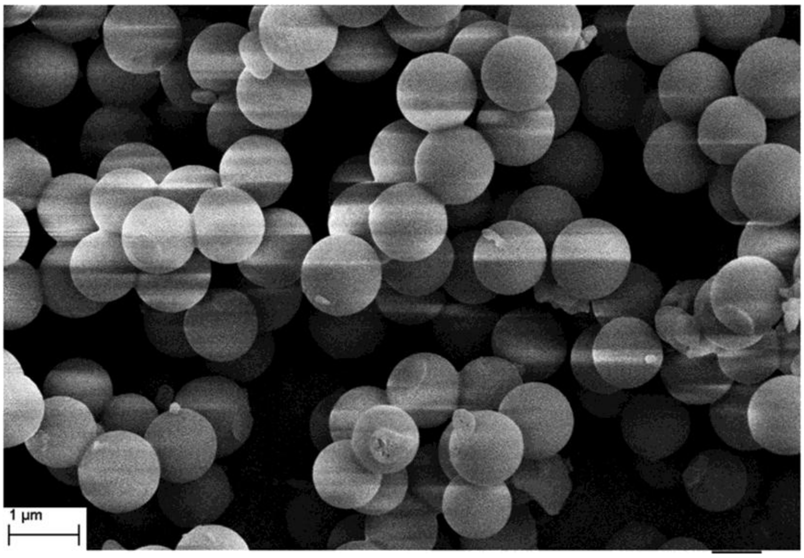

Figure S21. SEM images of the precipitate of **MH** at 2 h.

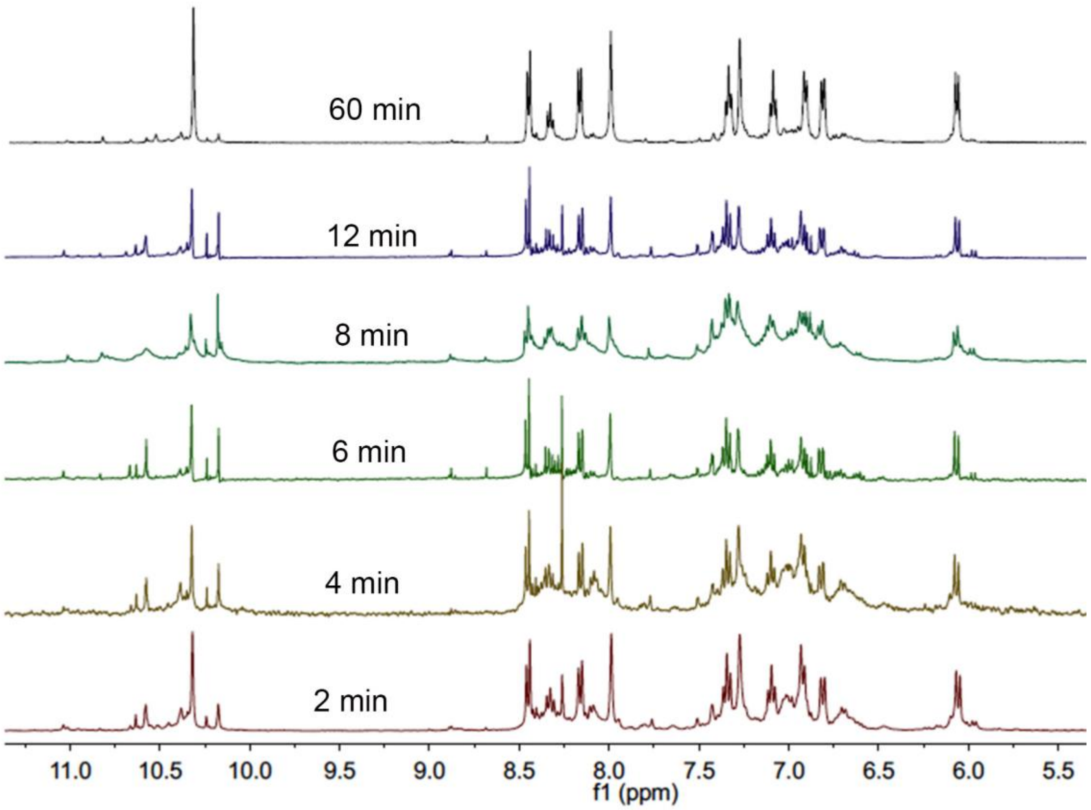

Figure S22. ^{1}H NMR spectra (400 MHz, d_6-DMSO) of the precipitates which were produced at different reaction times

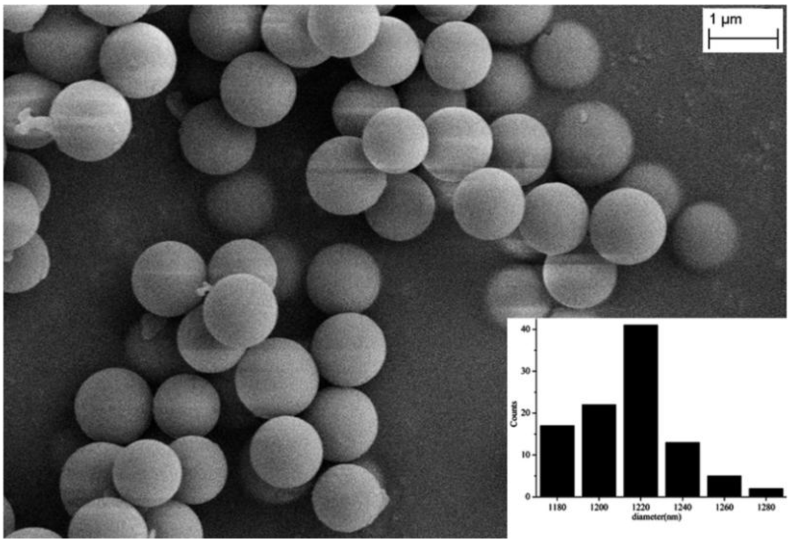

(a)

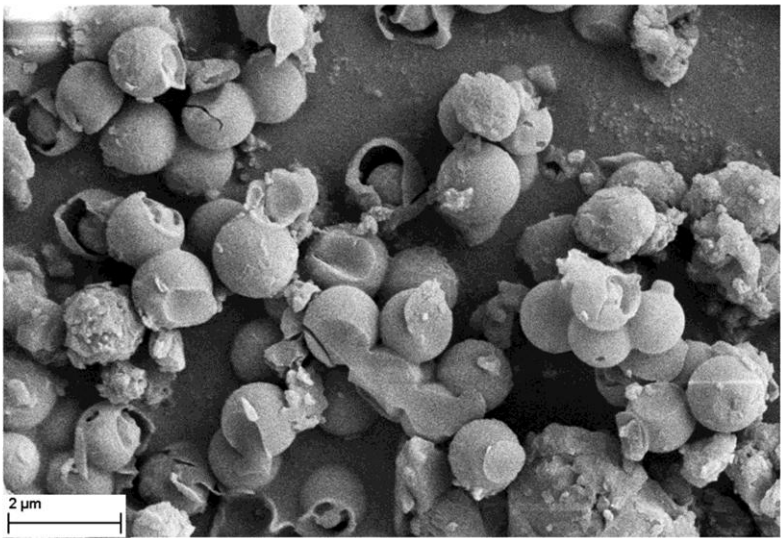

(b)

Figure S23. (a) SEM image and particle size distribution diagram of the formed microspheres of **MH** in the case of addition of 0 ml water to the reaction solution. (b) SEM image of the precipitate sample which was heated at 210 ^oC for 5 min.

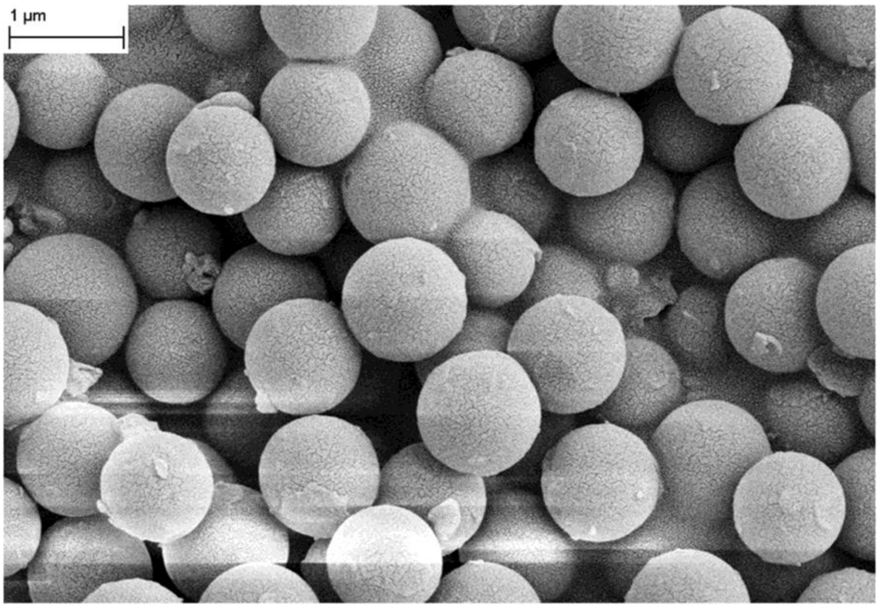

(a)

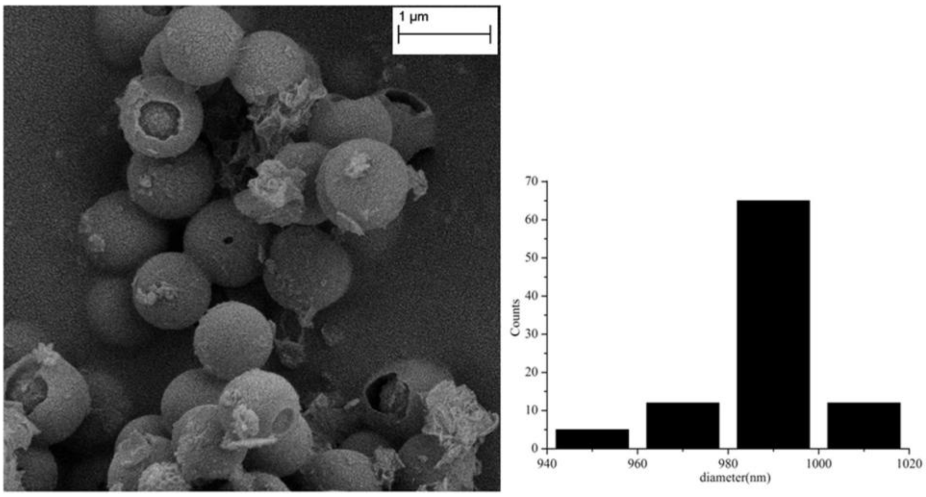

(b)

Figure S24. (a) SEM image and particle size distribution diagram of the formed microspheres of **MH** in the case of addition of 1.0 ml water to the reaction solution. (b) SEM image of the related precipitate sample which was heated at 210 ^{o}C for 5 min.

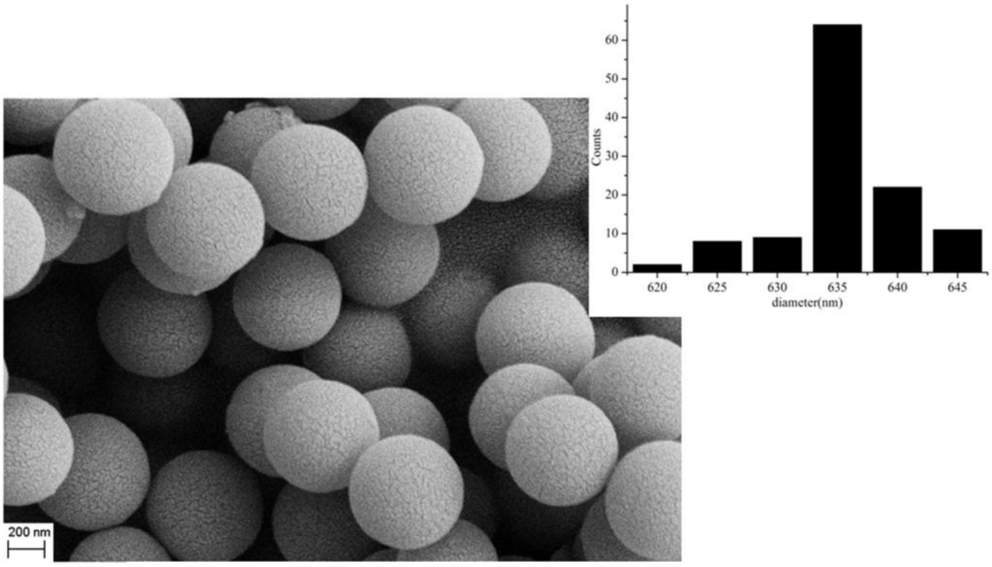

(a)

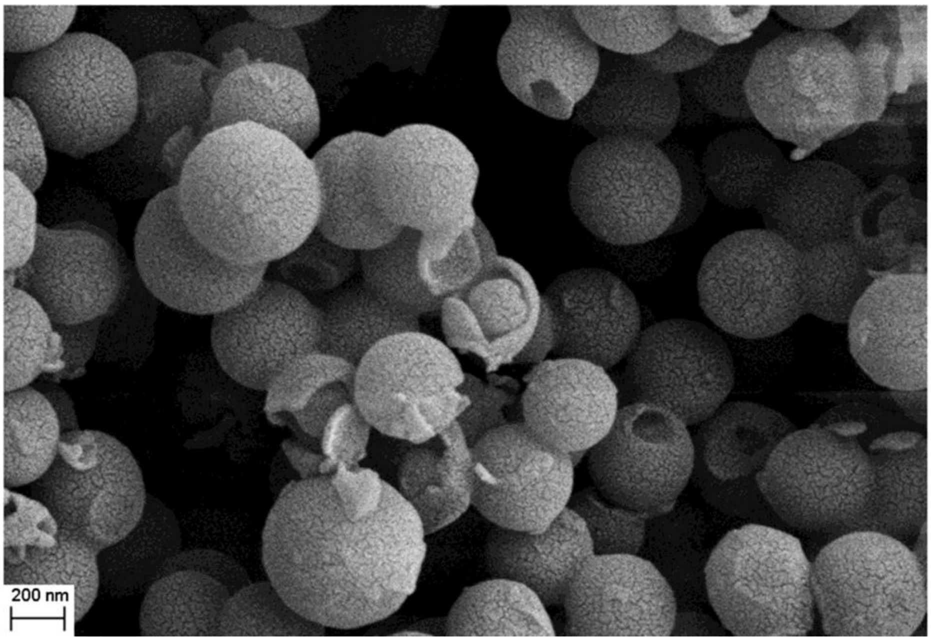

(b)

Figure S25. (a) SEM image and particle size distribution diagram of the formed microspheres of **MH** in the case of addition of 3.0 ml water to the reaction solution. (b) SEM image of the related precipitate sample which was heated at 210 ^{o}C for 5 min.

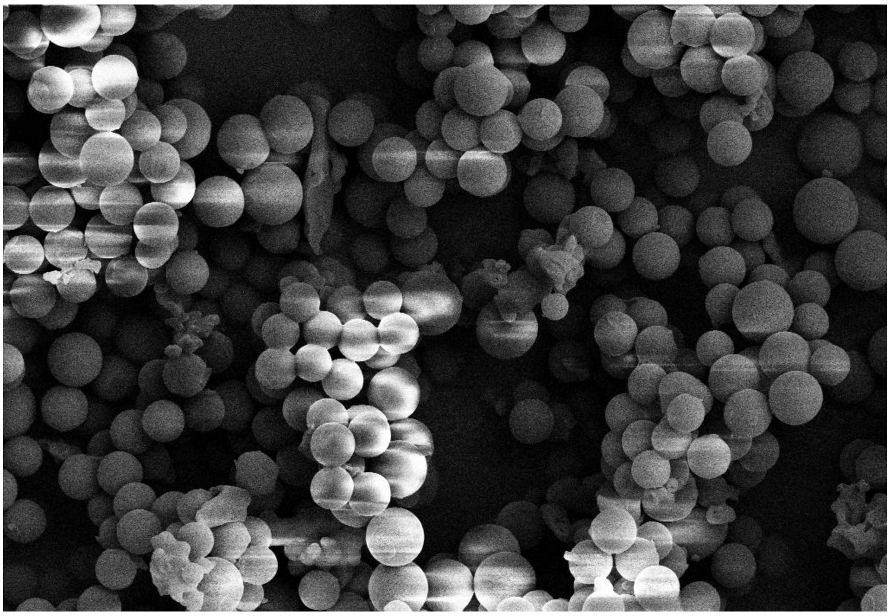

Figure S26. SEM image of the formed microspheres of **MH** in the case of addition of 0.5 ml water to the reaction solution.

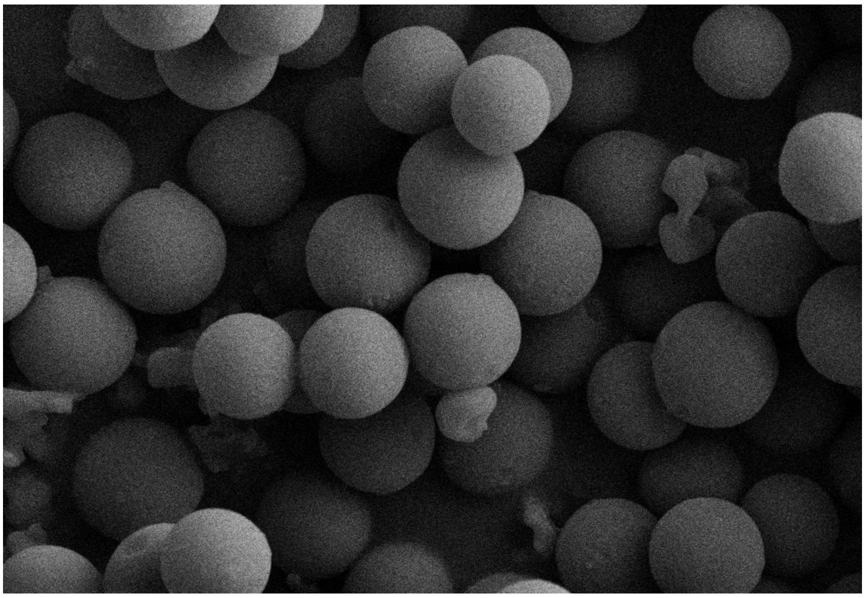

Figure S27. SEM image of the formed microspheres of **MH** in the case of addition of 2.0 ml water to the reaction solution.

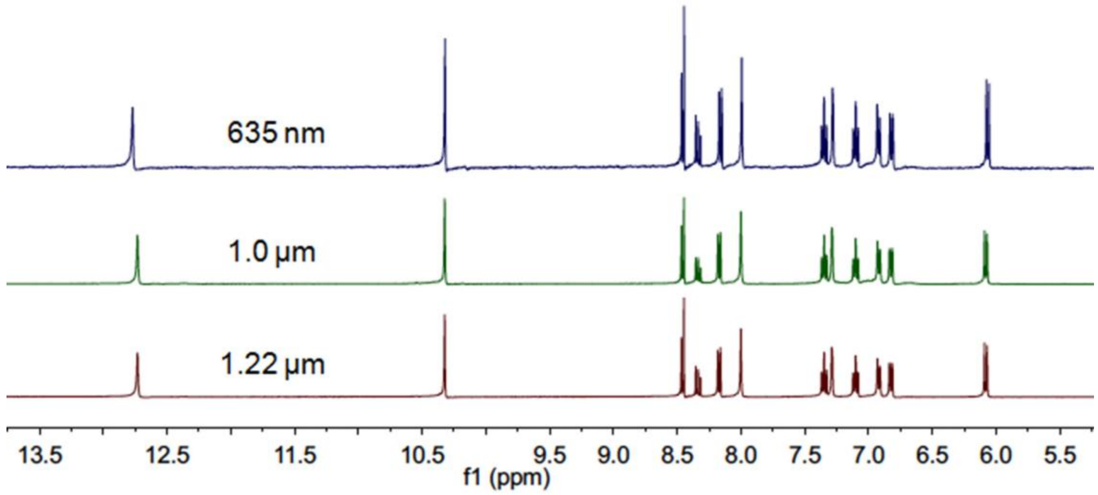

Figure S28. ^{1}H NMR spectra (400 MHz, d_6-DMSO) of the organic particles in different sizes.

Video. Substrates **1** (78 mg, 0.225 mmol) and **2** (64 mg, 0.225 mmol) were dissolved in methanol solution (20 mL) for 10 min., and 15 μL Conc. H_2SO_4 was added to the solution. The resulting mixture was stirred at room temperature and a light yellow precipitate was fast formed from the reaction solution.
